# Host plant associations in Western Palaearctic *Longitarsus* flea beetles (Chrysomelidae, Galerucinae, Alticini): a preliminary phylogenetic assessment

**DOI:** 10.3897/zookeys.856.32430

**Published:** 2019-06-17

**Authors:** Daniele Salvi, Paola D’Alessandro, Maurizio Biondi

**Affiliations:** 1 Department of Health, Life and Environmental Sciences, University of L’Aquila, Via Vetoio, 67100 Coppito, L’Aquila, Italy University of L’Aquila L’Aquila Italy; 2 CIBIO-InBIO, Centro de Investigação em Biodiversidade e Recursos Genéticos, Universidade do Porto, Campus Agrário de Vairão, 4485-661 Vairão, Portugal Universidade do Porto Vairão Portugal

**Keywords:** *
Longitarsus
*, molecular phylogeny, Palaearctic region, phylogenetic conservatism in host use, phytophagous insects

## Abstract

*Longitarsus* Latreille (Chrysomelidae, Galerucinae, Alticini) is a very large genus of phytophagous insects, with more than 700 species distributed in all zoogeographical regions. Patterns of host use have been a central topic in phytophagous insect research. In this study a first assessment is provided to test the hypothesis that host-plant association is phylogenetically conserved in Western Palaearctic *Longitarsus* species. Maximum Likelihood and Bayesian Inference methods were used to infer a phylogeny based on DNA sequence data from two mitochondrial genes from 52 *Longitarsus* species from the Western Palaearctic. In agreement with the host phylogenetic conservatism hypothesis, a strict association between most of the recovered clades and specific plant families was found, except for species associated with Boraginaceae. Low phylogenetic resolution at deep nodes limited the evaluation of whether closely related *Longitarsus* clades are associated with the same plant family or to closely related plant families.

## Introduction

*Longitarsus* Latreille, 1829 is a mega-diverse genus of phytophagous insects and the most speciose among flea beetles (Chrysomelidae, Galerucinae, Alticini) with more than 700 known species. It is widespread through all zoogeographical regions (Furth 2007, Biondi and D’Alessandro 2010, 2012, Döberl 2010, Prathapan and Viraktamath 2011, Reid 2017, unpublished data). *Longitarsus* is also ecologically diversified with specialized feeders, monophagous or oligophagous (Schoonhoven et al. 2005), on different angiosperm families. Larvae feed mostly on roots, and adults target leaves of their host plants (Dobler et al. 2000, Furth 1980). The monophyly of *Longitarsus* is accepted based on molecular evidence (Gómez-Rodríguez et al. 2015, Nie et al. 2018). Members of the genus are recognized mainly by the length of first metatarsomere, exceeding half-length of hind tibia, along with confuse elytral punctuation and absence of dorsal pubescence (Biondi and D’Alessandro 2012).

Relationships among Chrysomelidae and their host plants has been investigated from a biochemical, behavioural, and phylogenetic point of view, and at various taxonomic levels, often with the aim of understanding the biology of actual or potential pests (Jolivet and Hawkeswood 1995, Becerra and Vernable 1999, Dobler et al. 2000, Clark et al. 2004, Fernandez and Hilker 2007, Kergoat et al. 2007, Reid 2017). Understanding the mechanisms which drive observed host-use patterns has been a central topic in phytophagous insect research. Among the non-mutually exclusive hypotheses proposed (Gripenberg et al. 2010, Balagawi et al. 2013, Charlery de la Masselière et al. 2017, Kergoat et al. 2017, Lima Bergamini et al. 2017, Jones et al. 2019), the phylogenetic conservatism states that the phylogeny of host plants strongly constrains host affiliations. The phylogenetic conservatism hypothesis is widely demonstrated, even though it can be masked to varying degrees of convergent evolution in both plant and herbivore traits, and/or by phenotypic plasticity of both plants and herbivores; in addition, evolutionary processes that have generated phylogenetic conservatism patterns often remain unclear (Fernandez and Hilker 2007, Kergoat et al. 2017, Lima Bergamini et al. 2017). A comprehensive phylogenetic assessment of the insect-host plant relationship in the genus *Longitarsus* is still lacking, as well as comprehensive systematic studies on the genus.

In this paper, we conduct a molecular phylogenetic analysis on 52 Western Palaearctic *Longitarsus* species to map host plant data in order to assess whether the pattern observed is consistent with the phylogenetic conservatism hypothesis. If it is consistent, we would expect to find a) closely related *Longitarsus* species (single clades) to be associated with a specific plant family; b) *Longitarsus* clades associated with a specific plant family to form a single lineage.

## Methods

### Dataset, DNA extraction, amplification, and sequencing

We analysed 52 *Longitarsus* species (Table 1) from the Western Palaearctic, i.e., with distribution ranges centred on the Western Palaearctic area up to Urals, Caucasus, Anatolia, Iran, Near East, North Africa, and Macaronesia. Data on species distribution were mainly from Döberl (2010), and Gruev and Döberl (1997, 2005).

**Table 1.** Information on host plants, collection, and GenBank accession numbers for the 52 species of *Longitarsus* and *Batophilaaerata* (outgroup) used in this study.

Species	Host plant family	Trophic Range	Source for molecular analysis	Permits	Deposits	GenBank accession number	
						cox1	16S
*Batophilaaerata* (Marsham)	Rosaceae	OLI	Italy, Abruzzo (AQ), Roio Piano, 42°20'00.85"N 13°20'06.28"E, 930 m a.s.l., 13.v.2017, adult on *Rubus* sp. (Rosaceae) [det. M. Biondi, voucher db37]	not needed	Collection M. Biondi, University of L’Aquila (Italy)	requested	requested
*Longitarsusaeneicollis* (Faldermann)	Asteraceae, Boraginaceae, Lamiaceae	POL	Italy, Abruzzo (TE), Prati di Tivo, 42°30'26.40"N 13°33'14.46"E; 1344 m a.s.l., 11.vii.2018, adult on Boraginaceae [det. M. Biondi, voucher db46]	Gran Sasso e Monti della Laga National Park. Prot. n. 0007403/18	Collection M. Biondi, University of L’Aquila (Italy)	requested	requested
*Longitarsusaeneus* Kutschera	Boraginaceae	OLI	Italy, Abruzzo (TE), Guazzano, 42°44'03.6"N 13°38'49.8"E, 563 m a.s.l., 24.iv.2017, adult on *Boragoofficinalis* (Boraginaceae) [det. M. Biondi, voucher db15]	not needed	Collection M. Biondi, University of L’Aquila (Italy)	KX943357	requested
*Longitarsusanchusae* (Paykull)	Boraginaceae	OLI	Italy, Abruzzo (AQ), Aprati, 42°32'10.9"N 13°27'14.9"E, 862 m a.s.l., 19.v.2017, adult on *Anchusaitalica* (Boraginaceae) [det. M. Biondi, voucher db19]	Gran Sasso e Monti della Laga National Park. Prot. n. 0007804/17	Collection M. Biondi, University of L’Aquila (Italy)	KM448991	requested
*Longitarsusatricillus* (Linnaeus)	Asteraceae, Ranunculaceae, Fabaceae, Scrophulariaceae	POL	GenBank			KX943363	KP763161
*Longitarsusballotae* (Marsham)	Lamiaceae	OLI	GenBank			KM446857	KC186008
*Longitarsusbedelii* Uhagon	?	?	GenBank			–	KP763150
*Longitarsusbrisouti* Heikertinger	Asteraceae	OLI	GenBank			KM439696	–
*Longitarsusbrunneus* (Duftschmid)	Ranunculaceae	MON	GenBank			KM452511	–
*Longitarsuscelticus* Leonardi	Lamiaceae	OLI	GenBank			KU908777	–
*Longitarsuscerinthes* (Schrank)	Boraginaceae	OLI	GenBank			KX943478	KX943478
*Longitarsuscurtus* (Allard)	Boraginaceae	OLI	GenBank			KX943501	KX943501
*Longitarsusdorsalis* (Fabricius)	Asteraceae	MON	GenBank			KX943359	KX943359
*Longitarsusechii* (Koch)	Boraginaceae	OLI	GenBank			KM439361	–
*Longitarsusexsoletus* (Linnaeus)	Boraginaceae	OLI	GenBank			KX943418	KX943418
*Longitarsusfallax* Weise	Boraginaceae	OLI	Italy, Abruzzo (TE), Piancarani, 42°44'30.6"N 13°43'58.2"E, 191 m a.s.l., 24.iv.2017, adult on *Boragoofficinalis* (Boraginaceae) [det. M. Biondi, voucher db14]	not needed	Collection M. Biondi, University of L’Aquila (Italy)	requested	requested
*Longitarsusfoudrasi* Weise	Scrophulariaceae	OLI	Italy, Abruzzo (AQ), Casaline, 42°25'01.4"N 13°11'58.2"E, 1126 m a.s.l., 8.vii.2018, adult on *Scrophular*ia sp. (Scrophulariaceae) [det. M. Biondi, voucher db43]	not needed	Collection M. Biondi, University of L’Aquila (Italy)	requested	requested
*Longitarsushelvolus* Kutschera	Lamiaceae	OLI	GenBank			KU916133	–
*Longitarsusholsaticus* (Linnaeus)	Plantaginaceae	OLI	GenBank			KJ965710	–
*Longitarsusisoplexidis* Wollaston	Boraginaceae	MON	Portugal, Madeira Island, Encumeada, 32°44'24.2"N 17°02'55.0"W, 1316 m a.s.l., 1.iii.2017, adult on *Echiumcandicans* (Boraginaceae) [det. M. Biondi, voucher db7]	not needed	Collection M. Biondi, University of L’Aquila (Italy)	requested	requested
*Longitarsuslanguidus* Kutschera	Asteraceae	MON	GenBank			KU906343	–
*Longitarsuslateripunctatus* (Rosenhauer)	Boraginaceae	OLI	Italy, Lazio (RM), Rome, 41°52'45.62"N 12°27'31.96"E, 50 m a.s.l., 7.v.2017, adult on *Echiumvulgare* (Boraginaceae) [det. M. Biondi, voucher db16]	not needed	Collection M. Biondi, University of L’Aquila (Italy)	requested	requested
*Longitarsuslewisii* (Baly)	Plantaginaceae	OLI	GenBank			KJ964861	–
*Longitarsuslindbergi* Madar & Madar	Asteraceae	OLI	Portugal, Madeira Island, Encumeada, 32°44'24.2"N 17°02'55.0"W, 1316 m a.s.l., 1.iii.2017, adult on *Pericallisaurita* (Asteraceae) [det. M. Biondi, voucher db23]	not needed	Collection M. Biondi, University of L’Aquila (Italy)	requested	requested
*Longitarsuslinnaei* (Duftschmid)	Boraginaceae	OLI	Italy, Abruzzo (AQ), Onna, 42°19'22"N 13°28'24"E; 650 m a.s.l., 14.iv.2017, adult on *Symphytumtuberosum* (Boraginaceae) [det. M. Biondi, voucher db12]	not needed	Collection M. Biondi, University of L’Aquila (Italy)	requested	requested
*Longitarsuslycopi* (Foudras)	Lamiaceae	OLI	Italy, Abruzzo (TE), Prato Selva, 42°31'46.74"N 13°30'19.08"E, 1178 m a.s.l., 11.vii.2018, adult on *Mentha* sp. (Lamiaceae) [det. M. Biondi, voucher db50]	Gran Sasso e Monti della Laga National Park. Prot. n. 0007403/18	Collection M. Biondi, University of L’Aquila (Italy)	KX943332	requested
*Longitarsusmelanocephalus* (De Geer)	Plantaginaceae	MON	GenBank			KX943469	KX943469
*Longitarsusmembranaceus* (Foudras)	Lamiaceae	OLI	GenBank			KX943473	KX943473
*Longitarsusminusculus* (Foudras)	Lamiaceae	OLI	GenBank			KM442213	KP763142
*Longitarsusnasturtii* (Fabricius)	Boraginaceae	OLI	Italy, Lazio (RM), Rome, 41°54'50.58"N 12°22'57.12"E, 65 m a.s.l., 14.v.2017 [det. M. Biondi, voucher db34]	not needed	Collection M. Biondi, University of L’Aquila (Italy)	KM442304	requested
*Longitarsusniger* (Koch)	Plantaginaceae	OLI	GenBank			KX943504	KX943504
*Longitarsusnigrocillus* (Motschulsky)	Convolvulaceae	OLI	GenBank			KX943464	KX943464
*Longitarsusnigrofasciatus* (Goeze)	Scrophulariaceae	OLI	GenBank			KX943438	KX943438
*Longitarsusobliteratus* (Rosenhauer)	Lamiaceae	OLI	Italy, Abruzzo (AQ), Roio Piano, 42°19'29.91"N 13°20'24.87"E, 1011 m a.s.l., 15.vii.2018, adult on *Thymus* sp. (Lamiaceae) [det. M. Biondi, voucher db57]	not needed	Collection M. Biondi, University of L’Aquila (Italy)	KM447410	requested
*Longitarsusochroleucus* (Marsham)	Asteraceae	OLI	GenBank			KM448633	KP763117
*Longitarsusordinatus* (Foudras)	Lamiaceae	OLI	GenBank			KX943383	KP763143
*Longitarsuspellucidus* (Foudras)	Convolvulaceae	OLI	GenBank			KR480207	–
*Longitarsuspinguis* Weise	Boraginaceae	OLI	Italy, Abruzzo (AQ), Campo Imperatore, 42°26'17.8"N 13°35'41.2"E, 1760 m a.s.l., 21.vi.2017, adult on *Cynoglossummagellense* (Boraginaceae) [det. M. Biondi, voucher db21]	Gran Sasso e Monti della Laga National Park. Prot. n. 0007804/17	Collection M. Biondi, University of L’Aquila (Italy)	requested	requested
*Longitarsusplantagomarittimus* Dollman	Plantaginaceae	MON	GenBank			–	EF421523
*Longitarsuspratensis* (Panzer)	Plantaginaceae	OLI	GenBank			KX943360	KX943360
*Longitarsuspulmonariae* Weise	Boraginaceae	OLI	GenBank			KU907407	–
*Longitarsusquadriguttatus* Pontoppidan	Boraginaceae	OLI	GenBank			KM446524	–
*Longitarsusrectilineatus* (Foudras)	Lamiaceae	OLI	Italy, Lazio (RI), Val di Fua, 42°10'30"N 13°19'19"E; 1300–1500 m a.s.l.,13.ix.2017, adult on *Daphnelaureo*la (Thymelaeaceae; refuge plant) [det. M. Biondi, voucher db35]	Sirente Velino Natural Park Prot. n. 1441 22/06/2017	Collection M. Biondi, University of L’Aquila (Italy)	requested	requested
*Longitarsusreichei* (Allard)	Plantaginaceae	OLI	GenBank			KU907319	–
*Longitarsusrutilus* (Illiger)	Scrophulariaceae	MON	GenBank			KX943491	KX943491
*Longitarsussalviae* Gruev	Lamiaceae	MON	Italy, Abruzzo (AQ), Ortolano, 42°29'51.3"N 13°23'12.5"E, 1133 m a.s.l., 27.vii.2017, adult on *Thymus* sp. (Lamiaceae) [det. M. Biondi, voucher db31]	Gran Sasso e Monti della Laga National Park. Prot. n. 0007804/17	Collection M. Biondi, University of L’Aquila (Italy)	KU907298	requested
*Longitarsussaulicus* Gruev & Doeberl	Boraginaceae	OLI	Israel, Har Bracha, Amasa Spring, 32°11'35.93"N 35°15'54.21"E, 864 m a.s.l., 27.iii.2017, adult on *Anchusa* sp. (Boraginaceae) [det. M. Biondi, voucher db11]	not needed	Collection M. Biondi, University of L’Aquila (Italy)	requested	requested
*Longitarsusscutellaris* (Mulsant & Rey)	Plantaginaceae	MON	GenBank			KR484199	–
*Longitarsusspringeri* Leonardi	Asteraceae	MON	Italy, Abruzzo (AQ), Campo Imperatore, 42°26'55"N 13°32'24"E, 2140 m a.s.l., 23.viii.2018, adult on *Seneciorupestris* (Asteraceae) [det. M. Biondi, voucher db24]	Gran Sasso e Monti della Laga National Park. Prot. n. 0007403/18	Collection M. Biondi, University of L’Aquila (Italy)	requested	requested
*Longitarsussuccineus* (Foudras)	Asteraceae	OLI	Italy, Abruzzo (AQ), Valico delle Capannelle, 42°27'33.96"N 13°21'7.56"E, 1312 m a.s.l., 15.v.2017 [det. M. Biondi, voucher db53]	Gran Sasso e Monti della Laga National Park. Prot. n. 0007804/17	Collection M. Biondi, University of L’Aquila (Italy)	KJ962137	requested
*Longitarsussuturellus* (Duftschmid)	Asteraceae	OLI	GenBank			KU915636	–
*Longitarsustabidus* (Fabricius)	Scrophulariaceae	OLI	GenBank			KX943424	KX943424
*Longitarsuszangherii* Warchalowski	Asteraceae	MON	Italy, Abruzzo (TE), Prati di Tivo, 42°30'26.40"N 13°33'14.46"E, 1344 m a.s.l., 11.vii.2018, adult on *Petasites* sp. (Asteraceae) [det. M. Biondi, voucher db47]	Gran Sasso e Monti della Laga National Park. Prot. n. 0007403/18	Collection M. Biondi, University of L’Aquila (Italy)	requested	requested

To assess the association between phylogeny and patterns of host use, we used information on host plants for each species of *Longitarsus* from field observations over many years, and from a critical analysis of information reported in the literature (Furth 1980, Doguet 1994, Biondi 1995a, b, 1996, Bienkovski 2004, Konstantinov 2005, Aslan and Gok 2006, Gruev and Tomov 2007). We ignored isolated observations or reports of one or a few individual herbivores seen only one time on a host, as well as cases of post-season host refugium (Furth 1980). According to Biondi (1996), species feeding on one or two phylogenetically very closely related plant genera were considered as monophagous (**MON**); species feeding on one or two phylogenetically very closely related plant families were considered as oligophagous (**OLI**); species feeding on more plant species not phylogenetically closely related were considered as polyphagous (**POL**).

Phylogenetic relationships among plant families were discussed according to The Angiosperm Phylogeny Group 2016 (hereafter The APG 2016). Molecular analyses included DNA sequences from the mitochondrial genes cytochrome oxidase subunit I (cox1) and 16S rDNA (16S). These sequences were obtained from 20 alcohol-preserved specimens, each representing a distinct species, and retrieved from GenBank for 32 species (see Table 1 for information on specimens). We selected these two genes because they are the most represented in GenBank for *Longitarsus* species and because their phylogenetic utility at the genus level have been demonstrated by Nie et al. (2018). Details on sample data, along with GenBank accession numbers are provided in Table 1.

Total genomic DNA was extracted from preserved specimens using either a standard high-salt protocol (Sambrook et al. 1989) or the DNeasy Blood & Tissue extraction kit (Qiagen, Hilden, Germany), following the manufacturer’s protocol. Polymerase chain reaction (PCR) ampliﬁcation were performed using the universal primers LCO1490 and HC02198 (Folmer et al. 1994) and the primers specifically designed in this study for *Longitarsus* LonLCO-F (5’-CTC AGC CAT TTT ACC GAA TAA ATG-3’) and LonHCO-R (5’-GGA TTT GGI ATA ATT TCY CATA TTG-3’) targeting the barcoding fragment of the cox1 gene, and the primers 16sar-L (5-CGCCTGTTTATCAAAAACAT-3) and 16sbr-H (5’-CCG GTC TGA ACT CAG ATC AC-3’) slightly modified in Bologna et al. (2005) after Palumbi et al. (1991) targeting the fragment encompassing the domains IV and V of the16S rDNA. Amplification was carried out in a total volume of 25μl, with 3μl of PCR buffer, 2–2.5 μl of MgCl_2_ (50 mM), 0.5 μl of each primer (10mM), 0.5 μl of BSA, 1 U of BIOTAQ DNA polymerase (Bioline Ltd, London, UK) and 0.5–1 µL DNA template. PCR cycling conditions for cox1 followed Salvi et al. (2018), for 16S were 3 min at 94 °C, 35 cycles of 60 s at 94 °C, 90 s at 49.5 °C, 90 s at 72 °C, 10 min at 72 °C for final extension. Purification and sequencing of PCR products were carried out by an external service (Genewitz, UK).

### Phylogenetic analyses

Multiple sequence alignment was performed with MAFFT v.7 (Katoh and Standley 2013) using the E-INS-i iterative refinement algorithm. Phylogenetic analyses were conducted using Maximum Likelihood (ML) and Bayesian Inference (BI) approaches on the concatenated alignment of cox1 and 16S sequences, using as outgroup *Batophilaaerata* (Marsham, 1802). This outgroup belongs to *Altica* group, and has been demonstrated as a closely related lineage to *Longitarsus* group based on molecular evidence by Nie et al. (2018). Maximum Likelihood analyses were performed in raxmlGUI 1.5b2 (Silvestro and Michalak 2012), a graphical front‐end for RAxML 8.2.1 (Stamatakis 2014), with 10,000 rapid bootstrap replicates and 2000 independent ML searches (20% of the number of bootstrap replicates; command “-*f a*”; Stamatakis et al. 2008), applying the general time‐reversible model with a gamma model of rate heterogeneity (GTRGAMMA), with individual gene partitions.

Bayesian Inference analyses were performed in MrBayes 3.2.6 (Ronquist et al. 2012) using the best models of nucleotide substitution selected by JModelTest 2.1.1 (Darriba et al. 2012) under the corrected Bayesian Information Criterion (cox1: HKY+G; 16S: GTR+I+G). Two independent Markov chain Monte Carlo (MCMC) analyses with 6 chains each were run in parallel for 50 million generations, sampling every 5000 generations. The first 25% were discarded as burn-in. MCMC chains convergence was verified by average standard deviation of split frequencies values below 0.0035 and confirmed in Tracer 1.7 (Rambaut et al. 2018). A majority rule consensus tree with posterior probability of each node was calculated with the *sumt* command in MrBayes.

Phylogenetic results were summarised using the ML tree and reporting for each node both BS and BPP from the Bayesian analysis. Nodes with bootstrap values (BS) between 70 and 90% and Bayesian posterior probability (BPP) between 0.95 and 0.98 were considered as supported, and those with BS greater than 90% and BPP greater than 0.98 as highly supported.

## Results and discussion

**Figure 1.**
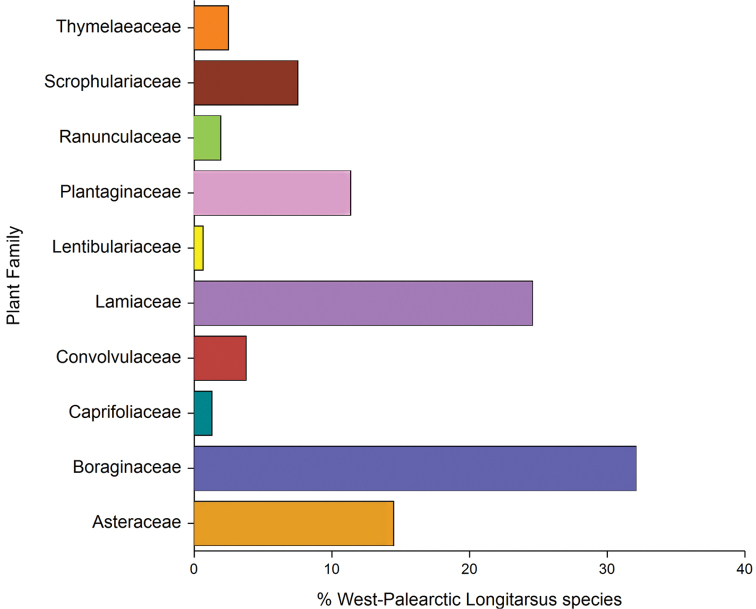
Percent distribution of oligophagous and monophagous Western Palaearctic species of *Longitarsus* on host plant families.

Host plants information is available for 165 out of 197 *Longitarsus* species known for the Western Palaearctic region. More than 96% of the species for which hosts are known, are specialized, either oligophagous or monophagous. The remaining species are generally considered as polyphagous (Fig. 1). Specialized feeders are distributed on plant families as follows (Fig. 1): Boraginaceae (51 species, 32.1%), Lamiaceae (39, 24.5%), Asteraceae (23, 14.5%), Plantaginaceae (18, 11.3%), Scrophulariaceae (12, 7.5%), Convolvulaceae (6, 3.8%), Thymeleaceae (4, 32.5%), Ranunculaceae (3, 1.9%), Caprifoliaceae (2, 1.3%), Lentibulariaceae (1, 0.6%).

Among the 52 *Longitarsus* species used in our molecular phylogenetic analyses (Table 1), 49 are monophagous or oligophagous; *L.atricillus* and *L.aeneicollis* are polyphagous, while no information is available about host plants of *L.bedelii*. Such a high number of specialized species allows a straightforward assessment of phylogenetic conservatism in host plant use, given a phylogenetic tree of the *Longitarsus* species and the relationships among host plant families.

**Figure 2.**
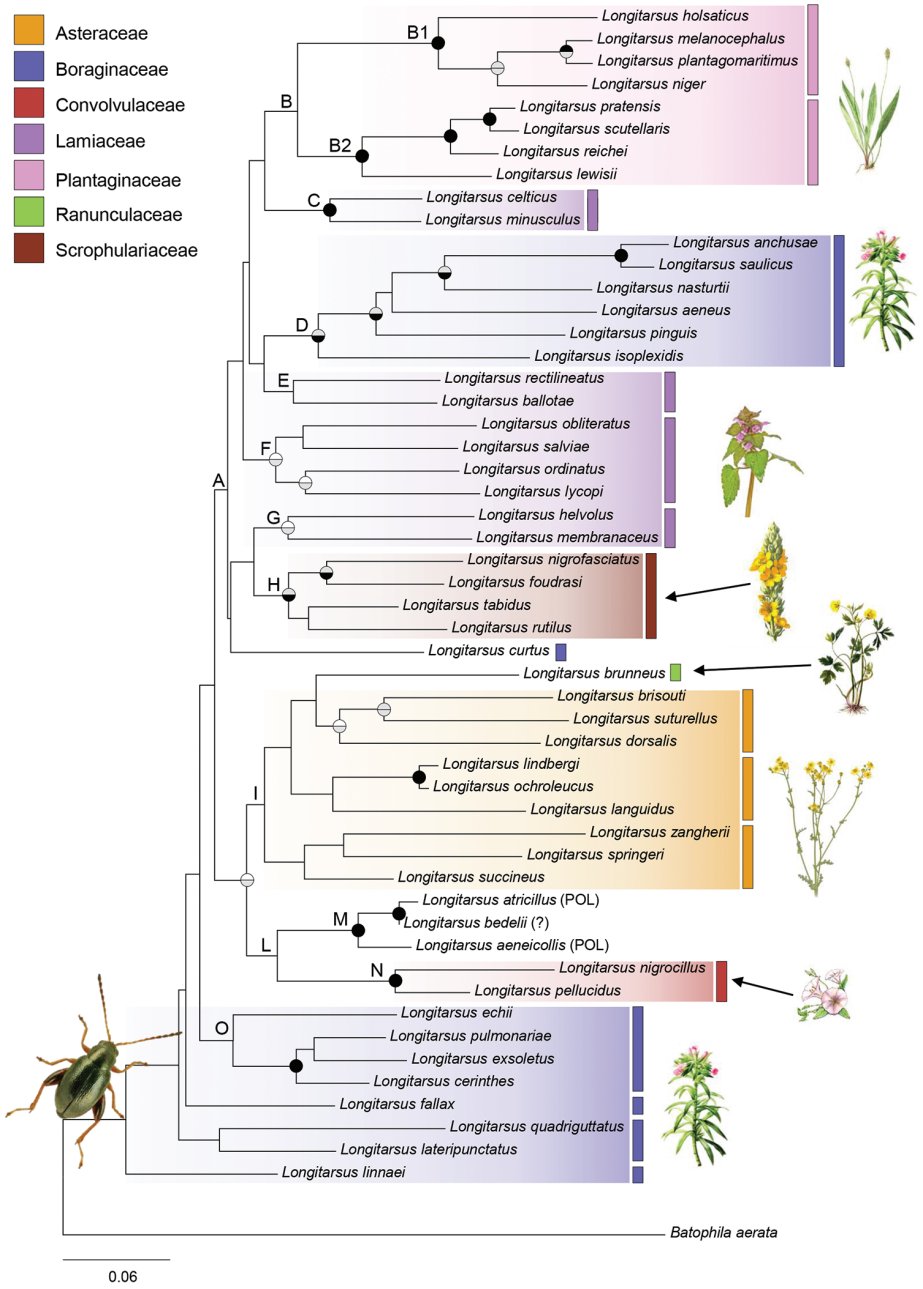
Maximum Likelihood phylogenetic tree of 52 species of *Longitarsus* based on concatenated cox1 and 16S DNA sequences. Circles in correspondence of nodes represent bootstrap support (BS, upper half) and posterior probability (BPP, bottom half) from Bayesian analysis: black for BS > 90 and BPP > 0.98; grey for BS of 70–90% and BPP of 0.95–0.98; white for BS of 50–70% only for nodes supported by Bayesian analysis. Abbreviations: POL = polyphagous; ? = host plants unknown.

Phylogenetic analyses based on ML and BI methods gave consistent results and identified the same supported clades (Fig. 2). Most of these clades have been previously recognized as distinct species-groups based on morphology (external morphology, aedeagus and/or spermatheca): (1) clade H includes species of the *tabidus* group sensu Leonardi (1972); (2) species of the *pratensis* group sensu Leonardi and Doguet (1990) are clustered in the clade B2; (3) *L.pulmonariae*, *L.exsoletus*, and *L.cerinthes* (clade O) were already known to be closely related (Leonardi 1972); (4) *L.anchusae* and *L.saulicus* (within clade D) belong to the *anchusae* group sensu Biondi (1995a).

In agreement with the host phylogenetic conservatism hypothesis, we recovered a strict association between most of the recovered *Longitarsus* clades and specific plant families, except for species associated with Boraginaceae (Fig. 2). *Longitarsus* species associated with Plantaginaceae form two closely related and supported clades (clades B1 and B2) and those associated with Scrophulariaceae form a distinct, well supported clade (clade H). Species associated with Lamiaceae are included in four clades (C, E, F, G) within a major lineage of Western Palaearctic *Longitarsus* (clade A). Relationships between clades within this lineage are poorly resolved; additional molecular data will be required to clarify relationships within clade A and to assess whether clades associated with Lamiaceae are truly polyphyletic or instead if increased phylogenetic resolution will allow recovering them as a monophyletic assemblage. All nine species associated with Asteraceae are grouped in the clade I; this clade also includes *L.brunneus* feeding on Ranunculaceae, and might represent an instance of host-shift towards an unrelated plant family (The APG 2016).

The two species associated with Convolvulaceae, *L.nigrocillus*, and *L.pellucidus*, cluster together with high support in clade N. The polyphagous species, *L.atricillus* and *L.aeneicollis*, plus *L.bedelii*, for which no host plants are known, form the highly supported clade M. On the other hand, clades grouping species associated with Boraginaceae are distant in the phylogenetic tree: clade D with six species and the isolated branch of *L.curtus* are included in clade A, whereas clade O with four species occupies a basal position of the phylogenetic tree together with other four species with poorly resolved phylogenetic position (*L.fallax*, *L.quadriguttatus*, *L.lateripunctatus*, *L.linnaei*).

Overall the phylogenetic tree of Western Palaearctic *Longitarsus* shows a decrease of statistical support from the tips to the root, with highly supported terminal clades and weakly supported basal relationships (Fig. 2). Therefore, the inference of phylogenetic conservatism in host-plant association is only robust at the lower hierarchical level. While the strong association between closely related *Longitarsus* species to the same plant family is clear, it is difficult to identify an association between closely related *Longitarsus* clades and closely related plant families. Most of the clades of *Longitarsus* (clades B-H) belonging to the same main lineage (clade A) are associated with plant families (Plantaginaceae, Scrophulariaceae, and Lamiaceae) that belong to the same order Lamiales (The APG 2016).

Ideally, for a conclusive assessment of phylogenetic conservatisms in host-plant association between closely related *Longitarsus* clades and closely related plant families we would require well-resolved phylogenies for both insects and plants at all taxonomic levels. While limited uncertainty exists for interrelationships between plant families (e.g., over the exact placement of Boraginaceae family (The APG 2016)), our molecular phylogenetic analysis only provides a first appraisal of relationships between Western Palaearctic *Longitarsus*. To assess whether the pattern of basal polytomy we observed (Fig. 2) is a solid polytomy or is due to a lack of data (see Mendes et al. 2016) further studies based on increased taxon and marker sampling are required. Improving field research is also crucial because the ecology and feeding biology of several species are still unknown. True host affiliation can be difficult to detect, due to the different interaction that phytophagous insects can have with plant species (Furth 1980, Schoonhoven et al. 2005), even though the use of molecular techniques, such as DNA barcoding of gut contents, can help to detect real trophic interactions (Jurado-Rivera et al. 2009).

## Conclusions

In this study, we provided first evidence that host-use patterns are phylogenetically constrained in Western Palaearctic *Longitarsus*. Despite the limited set of species analysed, we found a clear association between closely related *Longitarsus* species and specific plant families (Plantaginaceae, Asteraceae, Scrophulariaceae, and Convolvulaceae). However, relationships between clades of species were poorly resolved thus preventing the assessment of whether all *Longitarsus* clades associated with a specific plant family, or to related plant families, represent a single lineage. Such a relationship is unlikely for those *Longitarsus* species feeding on Boraginaceae which were resolved in unrelated clades. A better understanding of the phylogenetic relationships between *Longitarsus* species associated with Boraginaceae is of great interest also from a biogeographical point of view. In fact, two groups of species feeding on Boraginaceae and sharing a number of striking morphological features show a disjunct Mediterranean-South African distribution (Biondi 1995a, Biondi and D’Alessandro 2008, 2017). Molecular studies with additional markers are in progress on an extended set of species to further our understanding of hostplant relationships in *Longitarsus*.
